# The Anti-Circumsporozoite Antibody Response of Children to Seasonal Vaccination With the RTS,S/AS01_E_ Malaria Vaccine^[Author-notes ciab1017-FM1]^

**DOI:** 10.1093/cid/ciab1017

**Published:** 2021-12-11

**Authors:** Issaka Sagara, Issaka Zongo, Matthew Cairns, Rakiswendé Serge Yerbanga, Almahamoudou Mahamar, Frédéric Nikièma, Amadou Tapily, Frédéric Sompougdou, Modibo Diarra, Charles Zoungrana, Djibrilla Issiaka, Alassane Haro, Koualy Sanogo, Abdoul Aziz Sienou, Mahamadou Kaya, Seydou Traore, Ismaila Thera, Kalifa Diarra, Amagana Dolo, Irene Kuepfer, Paul Snell, Paul Milligan, Christian Ockenhouse, Opokua Ofori-Anyinam, Halidou Tinto, Abdoulaye Djimde, Jean Bosco Ouedraogo, Alassane Dicko, Daniel Chandramohan, Brian Greenwood

**Affiliations:** The Malaria Research and Training Center, University of Sciences, Techniques and Technologies, Bamako, Mali; Institut de Recherche en Sciences de la Santé, Bobo-Dioulasso, Burkina Faso; London School of Hygiene & Tropical Medicine, London, United Kingdom; Institut de Recherche en Sciences de la Santé, Bobo-Dioulasso, Burkina Faso; The Malaria Research and Training Center, University of Sciences, Techniques and Technologies, Bamako, Mali; Institut de Recherche en Sciences de la Santé, Bobo-Dioulasso, Burkina Faso; The Malaria Research and Training Center, University of Sciences, Techniques and Technologies, Bamako, Mali; Institut de Recherche en Sciences de la Santé, Bobo-Dioulasso, Burkina Faso; The Malaria Research and Training Center, University of Sciences, Techniques and Technologies, Bamako, Mali; Institut de Recherche en Sciences de la Santé, Bobo-Dioulasso, Burkina Faso; The Malaria Research and Training Center, University of Sciences, Techniques and Technologies, Bamako, Mali; Institut de Recherche en Sciences de la Santé, Bobo-Dioulasso, Burkina Faso; The Malaria Research and Training Center, University of Sciences, Techniques and Technologies, Bamako, Mali; Institut de Recherche en Sciences de la Santé, Bobo-Dioulasso, Burkina Faso; The Malaria Research and Training Center, University of Sciences, Techniques and Technologies, Bamako, Mali; The Malaria Research and Training Center, University of Sciences, Techniques and Technologies, Bamako, Mali; The Malaria Research and Training Center, University of Sciences, Techniques and Technologies, Bamako, Mali; The Malaria Research and Training Center, University of Sciences, Techniques and Technologies, Bamako, Mali; The Malaria Research and Training Center, University of Sciences, Techniques and Technologies, Bamako, Mali; London School of Hygiene & Tropical Medicine, London, United Kingdom; London School of Hygiene & Tropical Medicine, London, United Kingdom; London School of Hygiene & Tropical Medicine, London, United Kingdom; PATH, Seattle, Washington, USA; GSK Vaccines, Rixensart, Belgium; Institut de Recherche en Sciences de la Santé, Bobo-Dioulasso, Burkina Faso; The Malaria Research and Training Center, University of Sciences, Techniques and Technologies, Bamako, Mali; Institut de Recherche en Sciences de la Santé, Bobo-Dioulasso, Burkina Faso; The Malaria Research and Training Center, University of Sciences, Techniques and Technologies, Bamako, Mali; London School of Hygiene & Tropical Medicine, London, United Kingdom; London School of Hygiene & Tropical Medicine, London, United Kingdom

**Keywords:** anti-circumsporozoite antibody, RTS, S/AS01_E_ vaccine, seasonal vaccination, Mali, Burkina Faso

## Abstract

**Background:**

A trial in African children showed that combining seasonal vaccination with the RTS,S/AS01_E_ vaccine with seasonal malaria chemoprevention reduced the incidence of uncomplicated and severe malaria compared with either intervention given alone. Here, we report on the anti-circumsporozoite antibody response to seasonal RTS,S/AS01_E_ vaccination in children in this trial.

**Methods:**

Sera from a randomly selected subset of children collected before and 1 month after 3 priming doses of RTS,S/AS01_E_ and before and 1 month after 2 seasonal booster doses were tested for anti-circumsporozoite antibodies using enzyme-linked immunosorbent assay. The association between post-vaccination antibody titer and incidence of malaria was explored.

**Results:**

A strong anti-circumsporozoite antibody response to 3 priming doses of RTS,S/AS01_E_ was seen (geometric mean titer, 368.9 enzyme-linked immunosorbent assay units/mL), but titers fell prior to the first booster dose. A strong antibody response to an annual, pre-malaria transmission season booster dose was observed, but this was lower than after the primary vaccination series and lower after the second than after the first booster dose (ratio of geometric mean rise, 0.66; 95% confidence interval [CI], .57–.77). Children whose antibody response was in the upper tercile post-vaccination had a lower incidence of malaria during the following year than children in the lowest tercile (hazard ratio, 0.43; 95% CI, .28–.66).

**Conclusions:**

Seasonal vaccination with RTS,S/AS01_E_ induced a strong booster antibody response that was lower after the second than after the first booster dose. The diminished antibody response to the second booster dose was not associated with diminished efficacy.

**Clinical Trials Registration:**

NCT03143218.

## Teaser

Immunization of primed, young African children with a single dose of the RTS,S/AS01_E_vaccine prior to the malaria transmission season induced a rise in antibody titer to the *Plasmodium falciparum*circumsporozoite protein associated with substantial protection against *P. falciparum*malaria.

Malaria transmission is highly seasonal in 6 of the 10 African countries where malaria is not well controlled as identified by the World Health Organization [1]. Widespread deployment of seasonal malaria chemoprevention (SMC) has had a substantial impact on malaria in children in these areas [2]. However, in many parts of the Sahel and sub-Sahel, malaria remains the most frequent cause of death and hospital admission in young children [3]. Taking advantage of the high initial efficacy of the RTS,S/AS01_E_ malaria vaccine [4, 5], we have suggested that RTS,S/AS01_E_ could be deployed in these areas as a seasonal vaccine [6]. This concept has been tested in a trial undertaken in 5920 children in Burkina Faso and Mali during 2017–2020. Seasonal vaccination with RTS,S/AS01_E_ was noninferior to SMC in preventing clinical episodes of malaria, and the combination of RTS,S/AS01_E,_ with SMC was markedly superior to either intervention given alone in preventing uncomplicated cases of malaria, severe malaria requiring hospital admission, and death from malaria [7]. Here, we report on the anti-circumsporozoite (anti-CSP) antibody titers measured in a subset of trial children sampled before and after 3 priming doses of RTS,S/AS01_E_ and before and after 2 subsequent booster doses given just prior to the malaria transmission season, together with the correlation between anti-CSP antibody titer following vaccination and the incidence of episodes of uncomplicated clinical malaria during the subsequent year.

## METHODS

### Trial Design

Blood samples for serologic testing were collected during the course of an individually randomized, controlled trial designed to determine whether seasonal vaccination with the RTS,S/AS01_E_ malaria vaccine was noninferior to SMC in preventing clinical episodes of malaria and/or whether the combination was superior to either intervention given alone. The primary trial end point was the incidence of uncomplicated, microscopically confirmed *Plasmodium falciparum* malaria with a density of 5000 parasites per microliter or more. There were a number of additional secondary end points [8].

The 3 main objectives of the serologic substudy were determination of *P. falciparum* anti-CSP antibody titers before and after 3 priming doses of RTS,S/AS01_E_ and before and after 2 subsequent annual booster doses, whether the magnitude of the anti-CSP antibody response to priming or booster immunization influenced the risk of malaria during the subsequent malaria transmission season, and whether the anti-CSP antibody titer response to annual booster doses of RTS,S/AS01_E_ was influenced by administration of SMC in the previous malaria transmission season.

### Trial Sites and Population

The trial was conducted in Bougouni and Ouélessébougou districts, Mali, and in Houndé district, Burkina Faso. All households within the study areas with children aged 5–17 months on 1 April 2017 were enumerated in February 2017–March 2017. Eligible children whose parent or guardian provided consent for their child to join the trial were allocated randomly to an SMC alone, RTS,S/AS01_E_ alone, or RTS,S/AS01_E_ + SMC by an independent statistician.

### Interventions

**Figure 1.**
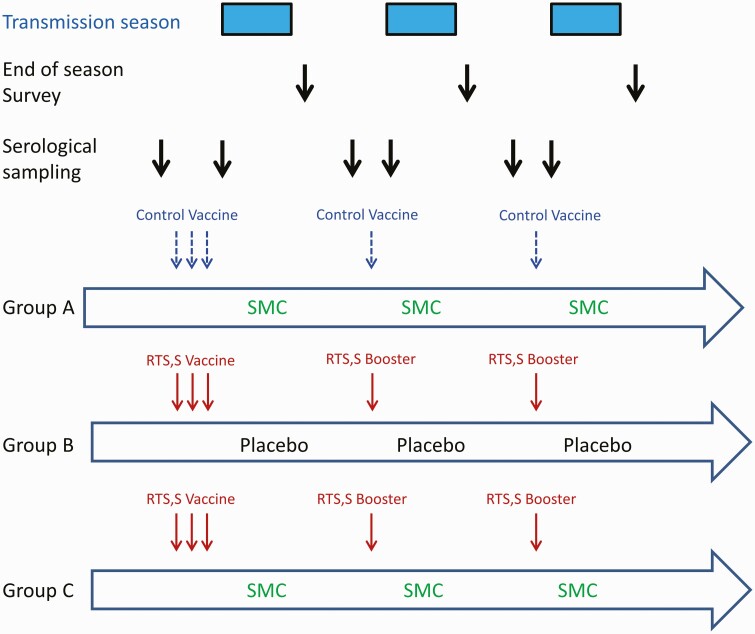
Schematic showing the interventions given to children in each of the 3 trial groups and their timing in relation to the malaria transmission seasons in 2017, 2018, and 2019. The timing of the collection of serological samples and of the cross-sectional surveys when samples were collected for malaria microscopy are also shown. Abbreviation: SMC, seasonal malaria chemoprevention.

Children in the RTS,S/AS01_E_ alone or RTS,S/AS01_E_ + SMC group received 3 doses of RTS,S/AS01_E_ vaccine (GSK, Rixensart, Belgium) at monthly intervals in April 2017–June 2017 followed by fourth and fifth doses in June 2018 and June 2019, just prior to the malaria transmission season (Figure 1). Children in the SMC alone group received 3 doses of rabies vaccine (Rabipur; Bavarian Nordic A/S, Denmark) in 2017 and a single dose of hepatitis A vaccine (HAVRIX; GSK, Rixensart, Belgium) in 2018 and 2019. The RTS,S/AS01_E_ + SMC and the SMC alone groups received 4 cycles of SMC at monthly intervals each year, while the RTS,S/AS01_E_ alone group received 4 cycles of SMC matching placebo. A course of SMC for a child aged >1 year comprised sulfadoxine/pyrimethamine (SP) 500/25 mg and amodiaquine (AQ) 150 mg on day 1 (Guilin Pharmaceuticals, Shanghai, China) and AQ 150 mg only on days 2 and 3. Infants received half of these doses. All doses were administered by project staff under direct observation. All study children were given an insecticide-treated bed net at enrollment in 2017.

### Surveillance for Malaria

Project staff based in study health facilities identified and treated all cases of malaria who presented at these facilities using a rapid diagnostic test and obtained a blood film for subsequent microscopy [8]. All hospital admissions of study children were documented by trial staff [8]. Blood films were read by 2 independent microscopists and, in instances of a discrepancy in positivity or density, by a third reader with discrepancies being resolved as described previously [9]. A cross-sectional survey of malaria prevalence was undertaken in all study children 1 month after the last round of SMC administration each year.

### Serology

In 2017, approximately 200 children (100 per group) and in 2018 and 2019 approximately 300 children (150 per group) from the RTS,S/AS01_E_ alone or RTS,S/AS01_E_ + SMC groups, together with 30–40 children from the SMC alone group, were selected at random by an independent statistician using systematic random sampling after sorting by age and sex to ensure that treatment groups were approximately balanced between these 2 variables. The same children were sampled pre- and post-vaccination within a study year, but different children were selected each year. The timing of the collection of blood samples in relation to vaccination is shown in Figure 1.

Immunoglobulin G anti-CSP antibody titers were measured using a standardized enzyme-linked immunosorbent assay (ELISA) at the CEVAC Laboratory, Ghent University, Belgium [10]. The lower limit of quantification for the assay was 1.9 ELISA units/mL (EU/mL), and samples with a titer below this lower limit (ie, titers with a value of 0) were assigned a titer of 0.95 EU/mL, half the lower limit of detection.

### Sample Size and Statistical Analyses

Based on the findings in previous RTS,S/AS01_E_ trials, large differences in anti-CSP titer between pre- and post-priming or booster vaccinations were anticipated. In order to determine whether prior administration of SMC influenced the immune response to booster vaccination, approximately 160 children who had received RTS,S/AS01_E_ with or without SMC were selected in 2018 and 2019 to give a study with 80% power to detect a difference of 25%–30% in GMT between children who had received SMC or placebo in the previous year.

Prespecified analyses, approved by the Data Safety and Monitoring Board (DSMB), included (a) calculation of the ratio of post-vaccination to pre-vaccination titers in each year of the study among children who had received the RTS,S/AS01_E_ vaccine; (b) comparison of the rise in titers after each booster dose; (c) comparison of the post-booster vaccination titers in children who had previously received SMC and those who had not; (d) comparison of the incidence of morbidity outcomes in relation to antibody response using Cox regression models with a robust standard error to account for multiple episodes per child and defining a potential cutoff titer associated with protection from the reverse cumulative plots [11]; and (e) comparison of the prevalence of *P. falciparum* infection at the end of the malaria transmission season in relation to antibody response using Poisson regression with a robust standard error. For comparisons (d) and (e), incidence and prevalence were compared between groups defined by terciles of anti-CSP titer.

### Ethics and Trial Oversight

The trial protocol was approved by the ethics committees of the London School of Hygiene & Tropical Medicine; the Ministry of Health, Burkina Faso; the University of Sciences, Techniques and Technologies of Bamako, Mali; and the national regulatory authorities of Burkina Faso and Mali. The DSMB reviewed serious adverse events, approved the statistical analysis plan, and archived the locked databases prior to unblinding. A steering committee gave scientific advice and monitored progress of the trial. Written, informed consent was obtained from the parents or guardians of all children in the trial.

## RESULTS

### Study Children

A total of 231 pre- and post-vaccination paired blood samples were obtained from study children in 2017, 202 from children who received RTS,S/AS01_E_ and 29 from children who received a control vaccine. In 2018, pre- and post-vaccination samples were obtained from 317 children, 279 from children who received RTS,S/AS01_E_ and 38 from children who received a control vaccine. In 2019, 327 pre- and post-vaccination samples were obtained, 291 from children who received RTS,S/AS01_E_ and 36 from children who received a control vaccine. The mean age and gender of children who were sampled in each study year are shown by study group in Table 1.

**Table 1.** Age, in Months, and Gender of the Pre-Vaccination Contacts in Each Year of the Study

Study Children\'s Characteristics	Contact	SMC Alone		RTS,S Alone		RTS,S + SMC		Both RTS,S Groups Combined	
		N	Mean (SD), %	N	Mean (SD), %	N	Mean (SD), %	N	Mean (SD), %
Both countries									
Age, Mean (SD)	Pre-2017	29	13.3 (4.17)	102	12.2 (4.38)	100	12.1 (4.22)	202	12.2 (4.29)
Sex, Percent (Male)		11	37.9	50	49.0	48	48.0	98	47.2
Age, Mean (SD)	Pre-2018	38	24.6 (4.55)	141	25.4 (4.22)	138	24.9 (4.18)	279	25.1 (4.20)
Sex, Percent (Male)		17	44.7	73	51.8	74	53.6	147	51.7
Age, Mean (SD)	Pre-2019	36	36.9 (4.30)	153	36.7 (3.93)	138	36.7 (3.99)	291	36.7 (3.95)
Sex, Percent (Male)		21	58.3	72	47.1	65	47.1	137	48.3
Burkina Faso									
Age, Mean (SD)	Pre-2017	17	14.1 (3.97)	43	12.6 (4.75)	44	12.3 (4.28)	87	12.5 (4.49)
Sex, Percent (Male)		7	41.2	23	53.5	22	50.0	45	50.0
Age, Mean (SD)	Pre-2018	16	25.6 (4.86)	65	25.1 (4.32)	67	24.8 (4.27)	132	25.0 (4.28)
Sex, Percent (Male)		8	50.0	35	53.8	38	56.7	73	54.7
Age, Mean (SD)	Pre-2019	17	36.9 (4.88)	71	37.2 (4.14)	64	37.0 (4.32)	135	37.1 (4.21)
Sex, Percent (Male)		9	52.9	34	47.9	27	42.2	61	46.1
Mali									
Age, Mean (SD)	Pre-2017	12	12.3 (4.38)	59	12.0 (4.12)	56	12.0 (4.20)	115	12.0 (4.14)
Sex, Percent (Male)		4	33.3	27	45.8	26	46.4	53	44.9
Age, Mean (SD)	Pre-2018	22	23.8 (4.28)	76	25.6 (4.16)	71	25.0 (4.13)	147	25.3 (4.14)
Sex, Percent (Male)		9	40.9	38	50.0	36	50.7	74	49.1
Age, Mean (SD)	Pre-2019	19	36.9 (3.84)	82	36.3 (3.72)	74	36.5 (3.70)	156	36.4 (3.70)
Sex, Percent (Male)		12	63.2	38	46.3	38	51.4	76	50.3

Abbreviations: SD, standard deviation; SMC, seasonal malaria chemoprevention.

### Baseline Anti-CSP Antibody Titers and the Anti-CSP Antibody Response to RTS,S/AS01_E_Vaccination

No child had an anti-CSP antibody titer above the lower limit of quantification (1.9 EU/mL) prior to vaccination. Two children in the SMC alone group who had not received RTS,S/AS01_E_ had a marked increase in anti-CSP antibody titer post-vaccination (post-vaccination GMTs 300.7 EU/mL and 728.2 EU/mL, respectively), probably resulting from a labeling error; these children were excluded from the analysis. With the exception of these 2 children, only 3 children in the SMC alone group had a titer above the lower limit of quantification at any survey.

**Figure 2.**
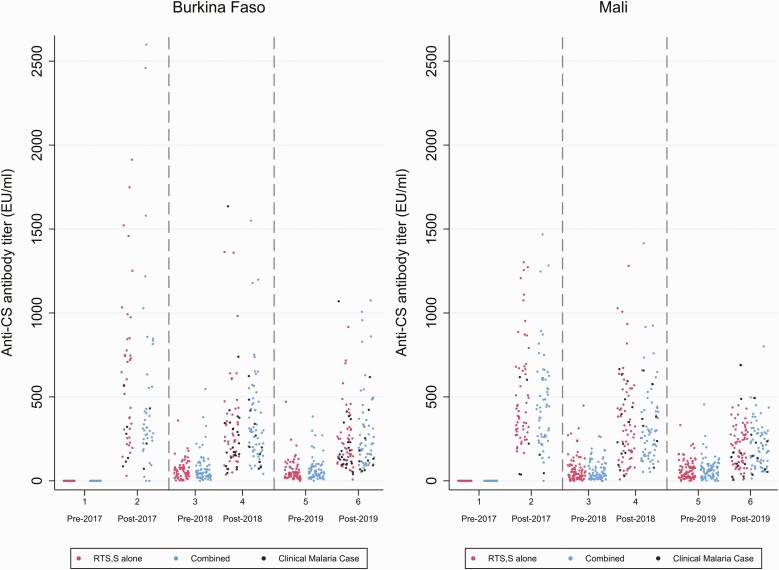
Anti-CSP antibody titers in individual children pre- and post-priming vaccination (2017) and pre and post first (2018) and second (2019) booster seasonal vaccination doses are shown by country. Results from children in the RTS,S/AS01_E_ alone group are shown in red, those from children in the combined group are shown in blue. Children who developed a clinical episode of malaria in the year after priming or booster vaccination are indicated in black. Abbreviations: CSP, circumsporozoite; EU, enzyme-linked immunosorbent assay unit.

**Figure 3.**
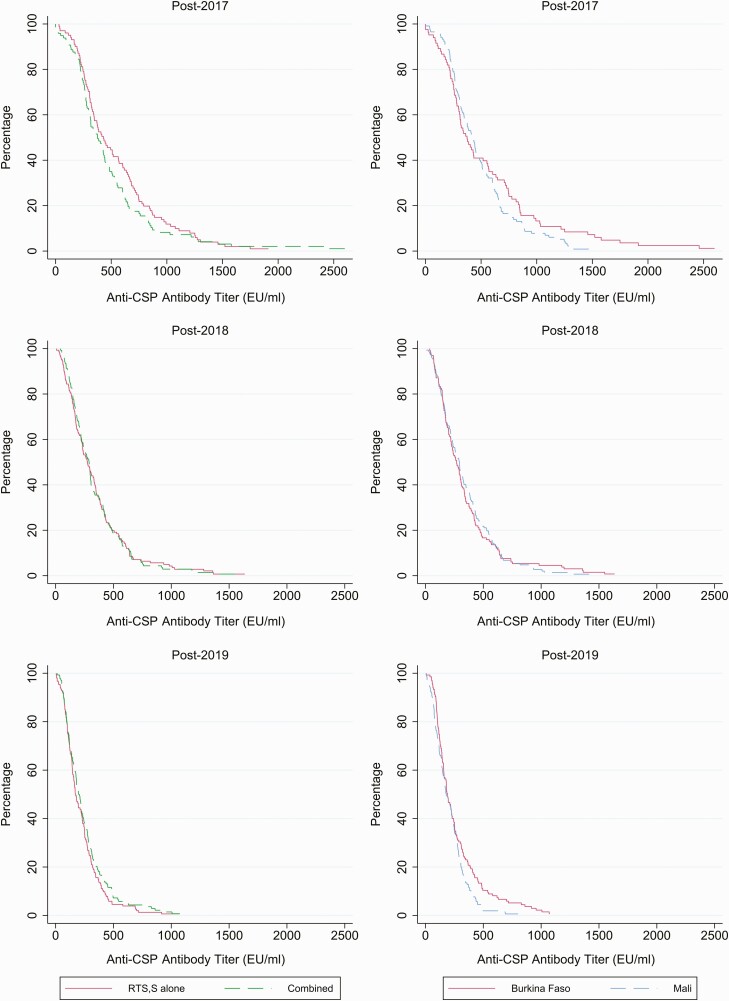
Reverse cumulative plots of antibody titer by study year, country, and study arm are shown. The top row shows post-vaccination titers in 2017, the middle row shows post-vaccination titers in 2018, and the bottom row shows post-vaccination titers in 2019. The right-hand set of panels shows titers by study country—Burkina Faso (solid red line), Mali (dashed blue line). The left-hand set of panels shows titers by study group—RTS,S/AS01_E_ alone group (solid red line), combined intervention group (dashed green line). Abbreviations: CSP, circumsporozoite; EU, enzyme-linked immunosorbent assay unit.

Among children in the RTS,S/AS01_E_ alone or RTS,S/AS01_E_ + SMC group, antibody titers increased markedly 1 month after the third of 3 priming doses, with a GMT of 368.9 EU/mL (95% confidence interval [CI], 317.7–428.4) being achieved (Table 2, Figures 2 and 3). The geometric mean ratio of post-vaccination–pre-vaccination titers was 446.5 (95% CI, 362.1–550.5). In children sampled in the second year, a year after priming, and immediately prior to administration of the first seasonal booster dose of RTS,S/AS01_E_, the GMT was 42.4 EU/mL (95% CI, 37.1–48.5). Following administration of the first booster dose (fourth dose), the GMT was 257.5 EU/mL (95% CI, 234.5–282.8) and the geometric mean ratio of post-booster titers–pre-booster titers was 5.81 (95% CI, 4.93–6.86). In children sampled immediately prior to the second booster, approximately 1 year following the first booster dose, the GMT was 44.4 EU/mL (95% CI, 39.2–50.1). Following the second booster dose, the GMT increased to 177.4 EU/mL (95% CI, 161.4–195.0) and the ratio between post-booster titers–pre-booster titers was 3.87 (95% CI, 3.40–4.41). The GMT following the first booster dose was significantly less than after the priming doses, and the GMT following the second booster dose was significantly lower than that seen after the first booster dose. The ratio of the geometric mean rise in titer following the second booster compared with the first booster was 0.66 (95% CI, .57–.77; Supplementary Table 1). Similar results were obtained in Burkina Faso and Mali. Responses to first or second booster doses in 2018 and 2019 were similar in children who had received SMC or SMC placebo in the previous year (Supplementary Table 2). Titers were generally lower in girls than in boys; this was most marked following the 2019 booster vaccination (ratio of GMC, 0.78; 95% CI, .65–.94; Supplementary Table 3).

**Table 2.** Anti-Circumsporozoite Antibody Titers Pre- and Post-Vaccination in Each Year in Children Who Received RTS,S/AS01_E_

Time of Sample Collection	N	Geometric Mean Titer, EU/mL (95% CI)	Geometric Mean Ratio Post- Vaccination–Pre-Vaccination (95% CI)	*P* Value	N With 2-Fold Increase in Titer (%)	N With 10-Fold Increase in Titer (%)
Both countries						
Pre-2017	201	0.95	[Ref]			
Post-2017	198	368.9 (317.7–428.4)	446.5 (362.1–550.5)	<.001	194/197 (98)	194/197 (98)
Pre-2018	279	42.4 (37.1–48.5)	[Ref]			
Post-2018	279	257.5 (234.5–282.8)	5.81 (4.93–6.86)	<.001	247/279 (89)	76/279 (27)
Pre-2019	291	44.4 (39.2–50.1)	[Ref]			
Post-2019	291	177.4 (161.4–195.0)	3.87 (3.40–4.41)	<.001	246/291 (85)	31/291 (11)
Burkina Faso						
Pre-2017	87	0.95	[Ref]			
Post-2017	83	349.7 (264.1–463.0)	464.9 (314.6–686.8)	<.001	81/83 (98)	81/83 (98)
Pre-2018	132	46.5 (38.6–56.0)	[Ref]			
Post-2018	132	256.3 (224.5–292.5)	5.40 (4.18–6.96)	<.001	111/132 (84)	31/132(23)
Pre-2019	135	43.6 (36.9–51.6)	[Ref]			
Post-2019	135	200.1 (175.6–228.0)	4.64 (3.88–5.55)	<.001	119/135 (88)	21/135 (16)
Mali						
Pre-2017	114	0.95	[Ref]			
Post-2017	115	383.5 (326.0–451.1)	433.6 (344.5–545.6)	<.001	113/114 (99)	113/114 (99)
Pre-2018	147	39.0 (32.1–47.4)	[Ref]			
Post-2018	147	258.6 (226.3–295.6)	6.20 (4.98–7.71)	<.001	136/147 (93)	45/147 (31)
Pre-2019	156	45.0 (37.7–53.8)	[Ref]			
Post-2019	156	159.9 (139.7–183.0)	3.31 (2.75–3.98)	<.001	127/156 (81)	10/156 (6)

Results from all children vaccinated with RTS,S/AS01_E_ (ie, RTS,S/AS01_E_ alone and combined groups pooled). At the pre-vaccination contact in 2017, 1 sample from Mali did not provide a conclusive result in the enzyme-linked immunosorbent assay assay. At the post-vaccination contact in 2017, 4 samples from Burkina Faso did not provide a conclusive result. There were no inconclusive results in subsequent years.

Abbreviations: CI, confidence interval; EU, enzyme-linked immunosorbent assay unit.

Nearly all children (>99%) showed a 10-fold increase in anti-CSP antibody titer 1 month after 3 priming doses, but this proportion fell to 27% after the first booster dose and to 11% after the second booster vaccination. The comparable percentages for a 2-fold increase in GMT were 89% and 85%, respectively (Table 2).

### Anti-CSP Titer and Protection Against Malaria

Terciles of the post-vaccination antibody response were defined separately for each year of the study (Table 3). Over the 3 years of the study combined, the hazard ratios comparing the incidence of clinical episodes of malaria between children in the highest or middle tercile with children in the lowest tercile were 0.43 (95% CI, .28–.66) and 0.66 (95% CI, .44–.99), respectively. Incidence was consistently lowest among children in the highest tercile in each year of the study. The hazard ratios for children in the highest tercile compared with those in the lowest tertile were 0.14 (95% CI, .03–.63), 0.71 (95% CI, .39–1.29), and 0.34 (95% CI, .18–.65) in years 1, 2, and 3, respectively.

**Table 3.** Incidence of Episodes of Clinical Malaria Overall and by Study Year in Children Included in the 2 Groups That Received RTS,S/AS01_E_, According to Anti-Circumsporozoite Antibody Titer Measured in the Post-Vaccination Sample

Anti-CSP Antibody Titer by Tercile	PYAR	Events	Rate per 1000 PYAR (95% CI)	Hazard Ratio (95% CI)	*P* Value
Overall					
Lowest	246.1	74	300.7 (239.4–377.6)	[Ref]	-
Medium	248.3	49	197.3 (149.1–261.1)	0.66 (0.44–0.99)	.044
Highest	248.4	31	124.8 (87.8–177.4)	0.43 (0.28–0.66)	<.001
2017					
Lowest (0.95–293.5 EU/mL)	59.4	12	202.0 (114.7–355.8)	[Ref]	-
Medium (297.6–570.6 EU/mL)	61.5	3	48.7 (15.7–151.1)	0.24 (0.07–0.90)	.034
Highest (601.0–2599.1 EU/mL)	60.1	2	33.3 (8.32–133.1)	0.14 (0.03–0.63)	.010
2018					
Lowest (8.2–185.6 EU/mL)	93.0	26	279.6 (190.4–410.6)	[Ref]	-
Medium (188.0–381.4 EU/mL)	91.9	28	304.5 (210.3–441.0)	1.11 (0.62–1.99)	.72
Highest (382.5–1635.4 EU/mL)	92.6	17	183.5 (114.1–295.2)	0.71 (0.39–1.29)	.26
2019					
Lowest (5.3–137.3 EU/mL)	93.7	36	384.1 (277.0–532.4)	[Ref]	-
Medium (137.5–263.3 EU/mL)	94.8	18	189.8 (119.6–301.3)	0.48 (0.25–0.90)	.022
Highest (266.5–1074.5 EU/mL)	95.7	12	125.4 (71.2–220.8)	0.34 (0.18, 0.65)	.001

Cox regression models for the pooled analysis over all 3 years of the study were adjusted for study year and the age of the child. The overall analysis aggregates person-time and events for the terciles defined separately in each year and children above and below the specific threshold in each year of the study.

Abbreviations: CI, confidence interval; EU, enzyme-linked immunosorbent assay unit; PYAR, person-years at risk.

Only 8 children in the serology substudy had an episode of malaria severe enough to require hospital admission; 3 after the first booster and 5 after the second booster. Three of these children had a post-vaccination titer in the lowest tercile, 2 in the middle, and 3 in the highest tercile.

The relationship between post-vaccination anti-CSP antibody titer 1 month after priming or after each booster vaccination and the prevalence of asymptomatic malaria parasitemia approximately 5 months later at the end of the malaria transmission season survey that year are shown in Table 4. In the first year of the study, only 3 malaria infections were detected among children in the serology substudy. However, this number increased to 27 and 22 in years 2 and 3, respectively. In year 2, the prevalence ratio for parasitemia for those whose post-booster vaccination titer was in the highest tercile compared with those whose titer was in the lowest tercile was 0.81 (95% CI, .33–2.0), and in year 3 (after the second booster), it was 0.40 (95% CI, .15–1.05). Identification of potentially protective cutoff titers in relation to the level of efficacy seen in each year of the study, deduced from reverse cumulative plots, gave values of 266.8 EU/mL, 207.2 EU mL, and 157.5 EU/mL in years 1, 2, and 3 of the study, respectively (Supplementary Table 4).

**Table 4.** Prevalence of *Plasmodium Falciparum* Infection at the End of Season Surveys Overall Which Were Conducted Approximately 1 Month After the Last Administration of SMC, and Approximately 5 Months After the Last Dose of Priming or the Booster Vaccine was Given, by Study Year

Anti-CSP Antibody Titer by Tercile	n/N	Prevalence (95% CI)	Prevalence Ratio (95% CI)	*P* Value
Overall				
Lowest	22/237	9.28 (6.19–13.7)	[Ref]	-
Medium	17/244	6.97 (4.37–10.9)	0.71 (.40–1.24)	.223
Highest	13/224	5.80 (3.40–9.75)	0.70 (.37–1.33)	.274
2017				
Lowest (0.95–293.5 EU/mL)	1/58	1.72 (.24–11.4)	[Ref]	
Medium (297.6–570.6 EU/mL)	0/62	0	0	-
Highest (601.0–2599.1 EU/mL)	2/54	3.70 (.92–13.8)	2.18 (.20–23.39)	.52
2018				
Lowest (8.2–185.6 EU/mL)	10/91	11.0 (6.00–19.3)	[Ref]	
Medium (188.0–381.4 EU/mL)	11/90	12.2 (6.88–20.8)	1.08 (.51–2.30)	.84
Highest (382.5–1635.4 EU/mL)	6/80	7.50 (3.40–15.8)	0.81 (.33–2.00)	.65
2019				
Lowest (5.3–137.3 EU/mL)	11/88	12.5 (7.04–21.2)	[Ref]	
Medium (137.5–263.3 EU/mL)	6/92	6.52 (2.95–13.8)	0.43 (.17–1.05)	.063
Highest (266.5–1074.5 EU/mL)	5/90	5.56 (2.32–12.7)	0.40 (.15–1.05)	.062

Poisson regression models for the pooled analysis over all 3 surveys were adjusted for study year and the age of the child. The overall analysis aggregates the number of children testing positive for *Plasmodium falciparum* and the total number of children for the tertiles defined separately in each year, and for children above and below the specific threshold in each year of the study.

Abbreviations: CI, confidence interval; EU, enzyme-linked immunosorbent assay unit.

## DISCUSSION

The anti-CSP antibody response of children in this trial to vaccination with 3 priming doses of RTS,S/AS01_E_ followed by a booster dose was similar to that seen in several previous studies in African children [12, 13]. However, it was lower than that recorded in the phase 3 RTS,S/AS01_E_ trial in which the GMT 1 month after 3 priming doses in those vaccinated between the ages of 5–17 months was 570.3 EU/mL (95% CI, 543.7–589.3) for all centers combined. A range of responses was seen between the 11 centers in the phase 3 trial with a high post-vaccination GMT of 686 EU/mL (95% CI, 604–778) being found at the Nanoro center in Burkina Faso [14, 15]. However, GMTs similar to those found in the present study were found in Lambarene, Gabon, and Lilongwe, Malawi. Half of the children in the current trial also received SMC. This is unlikely to have directly influenced the response to vaccination as this was given 1 month prior to SMC administration. Furthermore, there was no difference between the anti-CSP antibody response to booster immunization between children who had received SMC in the previous transmission season and those who had not. However, it is possible that reduction in the burden of malaria through administration of SMC enhanced the ability of RTS,S/AS01_E_ to induce more protective non–anti-CSP immune responses in the RTS,S/AS01_E_ combined group.

Relatively little is known about the impact of a booster dose of the RTS,S vaccine on either efficacy or the immune response. In the first clinical trial of RTS,S undertaken in Africa, a booster dose of RTS,S/AS02A given to adults 1 year after 3 priming doses gave an approximately 4-fold higher titer than that seen after priming and a partial return of protection against malaria infection [16]. However, in the phase 3 RTS,S/AS01_E_ trial in which a booster dose was given 18 months after priming, the post booster anti-CSP antibody GMT in children who entered the trial when aged 5–17 months was 318.2 EU/mL (95% CI, 295.1–343.0) [14], substantially less than that seen after priming. In the current trial, the response to a second booster dose was less than that seen after the first, raising a concern that further booster doses might result in a progressive decline in the immune response. A challenge study conducted in healthy adult volunteers showed a higher level of protection against challenge when a fractional dose of vaccine was given for the third priming and booster doses than in volunteers given 3 or 4 full doses [17], suggesting that the immunogenicity and efficacy of repeated seasonal vaccine booster doses of RTS,S/AS01_E_ might be improved if a fractionated dose was used. However, in a subsequent volunteer challenge study, no significant differences in efficacy were seen between groups in which various combinations of full and fractionated doses were explored [18].

The fact that the lower anti-CSP antibody response to a second booster dose compared with the first was not associated with any detectable loss of efficacy [7] is reassuring. It suggests that the second booster dose may have increased the quality of the antibodies produced in some way, for example, by altering their avidity and/or isotype, inducing protective antibodies to the C terminal of the CSP molecule, or antibodies binding to a Fcγ receptor promoting phagocytosis or activation of natural killer cells [19–27] or even to an increase in antibodies to blood stage antigens [28]. The RTS,S/AS01_E_ vaccine induces a strong cellular immune response [29–37], including CD4 immune responses associated with protection, and we hypothesize that repeated boosting might enhance T cell–mediated immune responses more markedly than that of the anti-CSP antibody responses. A systems biology approach is helping to characterize the relative importance of individual immune responses in the protection provided by RTS,S/AS01_E_ in human challenge studies [38, 39]. A weakness of this study is that it did not include cell-mediated immune assays; however, further serologic assays are now planned. Furthermore, children were also older after their second than after their first booster dose, and this could have influenced their overall immune response and influenced comparisons between years.

As noted in most, but not all, previous human challenge or field trials with RTS,S/AS01_E_, a correlation was found between anti-CSP antibody titer and incidence of clinical episodes of malaria. However, this association could be influenced by confounding, so this comparison is not sufficient to establish anti-CSP as a correlate of protection [40]. Moreover, the declining anti-CSP antibody response with successive boosters was not matched by a corresponding decline in efficacy, suggesting the potential protective role of other vaccine induced immunological responses, both humoral and cellular acting together.

In view of the encouraging efficacy results obtained with seasonal RTS,S/AS01_E_ vaccination given with SMC, the trial described here is being extended until children reach the age of 5 years, and the impact of third and fourth booster doses on the immune response and on efficacy against malaria is being followed.

## Supplementary Data

Supplementary materials are available at *Clinical Infectious Diseases* online. Consisting of data provided by the authors to benefit the reader, the posted materials are not copyedited and are the sole responsibility of the authors, so questions or comments should be addressed to the corresponding author.

ciab1017_suppl_Supplementary_Tables

## Notes

***Acknowledgments.*** The authors thank the members of the Trial Steering Committee (Feiko ter Kuile [chair], Kwadwo Koram, Mahamadou Thera, Joaniter Nankabirwa, and Morven Roberts) and the members of the Data and Safety Monitoring Board (Blaise Genton [chair], Sheick Coulibaly, Umberto D’Alessandro, Francesca Little, and Malcolm Molyneux) for their overview and support. They thank Sibyl Couvent, Sofie Librecht, Peter Vander Linden, and Gwenn Waerlop at Center for Vaccinology (CEVAC), the University of Ghent, for performing the laboratory work; Lode Schuerman, Geert Leroux-Roels, Frédéric Clement, and Katie Ewer for their helpful comments on the manuscript; Simon Correa and Mamadou Ndiath at the Medical Research Centre (MRC) Unit, The Gambia at the London School of Hygiene & Tropical Medicine, for performing the quality control on malaria blood film reading; Karen Slater for supporting the trial in many ways; GlaxoSmithKline Biologicals SA for donating the RTS,S/AS01_E_ and HAVRIX vaccines; Birkhäuser + GBC AG, Switzerland, for supplying identity cards and labels; Guilin Pharmaceuticals for supplying Seasonal Malaria Chemoprevention (SMC) drugs; the Ministry of Health staff in the Bougouni, Ouelessebougou, and Houndé districts for their assistance with running the trial; and all of the caretakers and children for their participation. Finally, they thank the late Ogobara Doumbo for helping to set up the trial.

***Financial support.*** The trial was funded by the UK Joint Global Health Trials (Department of Health and Social Care, Department for International Development, Global Challenges Research Fund, Medical Research Council, and Wellcome Trust; grant MR/P006876/1), with additional funding from PATH MVI (grant 18269). M. C. received support from an award (MR/R010161/1) jointly funded by the UK Medical Research Council (MRC) and the UK Department for International Development (DFID) under the MRC/DFID Concordat Agreement, which is also part of the European and Developing Countries Clinical Trials (EDCTP2) program supported by the European Union and is supported by a Sir Henry Dale Fellowship jointly funded by the Wellcome Trust and the Royal Society (grant 220658/Z/20/Z). N. F. reports that research grants were made to the London School of Hygiene & Tropical Medicine, United Kingdom, to Malaria Research and Training Center (MRTC), Mali, and to Institut de Recherche en Sciences de la Sante (IRSS), Burkina Faso, from the UK Joint Global Health Clinical Trials program and from PATH MVI to support the clinical trial on which this article is based.
