# Supplementary material for: The Anti-Circumsporozoite Antibody Response of Children to Seasonal Vaccination With the RTS,S/AS01E Malaria Vaccine
Source: Clin Infect Dis. 2021 Dec 11;75(4):613–22. doi: 10.1093/cid/ciab1017 (PMC9464075; doi:10.1093/cid/ciab1017)
Supplement: ciab1017_suppl_Supplementary_Tables [file ciab1017_suppl_supplementary_tables.docx]

**SUPPLEMENTARY TABLES**

Table S1. Comparison of the rise in vaccination titres after first and second booster doses in children included in the two groups who received RTS,S/AS01_E_ combined. In 2018, children received their first booster dose approximately one year after priming, in 2019 their second booster dose one year later.

|  | N Pre-booster titers | Geometric Mean pre-booster titer, EU/ml (95% CI) | N Post-booster titers | Geometric Mean post-booster titer, EU/ml (95% CI) | Geometric Mean rise in post -pre-booster titres | Ratio of Geometric Mean rise in titers (95% CI) | P value |
| --- | --- | --- | --- | --- | --- | --- | --- |
| **Both Countries** |  |  |  |  |  |  |  |
| Post-2018 | 279 | 42.4 (37.1, 48.5) | 279 | 257.5 (234.5, 282.8) | 6.07 (5.40, 6.83) | [Ref] | [Ref] |
| Post-2019 | 291 | 44.4 (39.2, 50.1) | 291 | 177.4 (161.4, 195.0) | 4.00 (3.64, 4.39) | 0.66 (0.57, 0.77) | <0.001 |
|  |  |  |  |  |  |  |  |
| **Burkina Faso** |  |  |  |  |  |  |  |
| Post-2018 | 132 | 46.5 (38.6, 56.0) | 132 | 256.3 (224.5, 292.5) | 5.51 (4.61, 6.58) | [Ref] | [Ref] |
| Post-2019 | 135 | 43.6 (36.9, 51.6) | 135 | 200.1 (175.6, 228.0) | 4.59 (4.03, 5.22) | 0.83 (0.67, 1.04) | 0.102 |
|  |  |  |  |  |  |  |  |
| **Mali** |  |  |  |  |  |  |  |
| Post-2018 | 147 | 39.0 (32.1, 47.4) | 147 | 258.6 (226.3, 295.6) | 6.63 (5.67, 7.76) | [Ref] | [Ref] |
| Post-2019 | 156 | 45.0 (37.7, 53.8) | 156 | 159.9 (139.7, 183.0) | 3.55 (3.11, 4.06) | 0.54 (0.44, 0.66) | <0.001 |

Table S2. Comparison of post-booster responses between children in the two intervention groups randomised to receive RTS,S/AS01_E_ combined, either with a placebo or active SMC starting one month after vaccination.

|  |  |  |  | Children with a two-fold increase in titer | | | Children with a ten-fold increase in titer | | | |
| --- | --- | --- | --- | --- | --- | --- | --- | --- | --- | --- |
|  | Arm | N | Geometric Mean post-booster titer, EU/ml (95% CI) | n | Prevalence ratio (95% CI) | P-value | n | Prevalence ratio (95% CI) | P-value |  |
| **Both Countries** | |  |  |  |  |  |  |  |  |  |
| Post-2018 | RTS,S alone | 141 | 247.1 (213.8, 285.6) | 122 | [Ref] |  | 33 | [Ref] |  |  |
|  | Combined | 138 | 268.7 (238.4, 302.7) | 125 | 1.05 (0.96, 1.14) | 0.29 | 33 | 1.33 (0.90, 1.96) | 0.15 |  |
|  |  |  |  |  |  |  |  |  |  |  |
| Post-2019 | RTS,S alone | 153 | 166.3 (144.8, 190.9) | 131 | [Ref] |  | 14 | [Ref] |  |  |
|  | Combined | 138 | 190.7 (167.7, 216.8) | 115 | 0.97 (0.88, 1.07) | 0.59 | 14 | 1.35 (0.69, 2.63) | 0.38 |  |
|  |  |  |  |  |  |  |  |  |  |  |
| **Burkina Faso** |  |  |  |  |  |  |  |  |  |  |
| Post-2018 | RTS,S alone | 65 | 242.3 (198.7, 295.5) | 54 | [Ref] |  | 13 | [Ref] |  |  |
|  | Combined | 67 | 270.6 (226.0, 324.0) | 57 | 1.02 (0.88, 1.19) | 0.75 | 13 | 1.34 (0.72, 2.52) | 0.36 |  |
|  |  |  |  |  |  |  |  |  |  |  |
| Post-2019 | RTS,S alone | 71 | 185.6 (154.4, 223.1) | 64 | [Ref] |  | 9 | [Ref] |  |  |
|  | Combined | 64 | 217.5 (180.1, 262.6) | 55 | 0.95 (0.84, 1.08) | 0.46 | 9 | 1.48 (0.67, 3.29) | 0.34 |  |
|  |  |  |  |  |  |  |  |  |  |  |
| **Mali** |  |  |  |  |  |  |  |  |  |  |
| Post-2018 | RTS,S alone | 76 | 251.2 (203.1, 310.7) | 68 | [Ref] |  | 20 | [Ref] |  |  |
|  | Combined | 71 | 266.8 (226.9, 313.8) | 68 | 1.07 (0.98, 1.17) | 0.15 | 20 | 1.34 (0.82, 2.19) | 0.25 |  |
|  |  |  |  |  |  |  |  |  |  |  |
| Post-2019 | RTS,S alone | 82 | 151.1 (123.2, 185.4) | 67 | [Ref] |  | 5 | [Ref] |  |  |
|  | Combined | 74 | 170.2 (142.8, 202.8) | 60 | 0.99 (0.85, 1.15) | 0.92 | 5 | 1.11 (0.33, 3.69) | 0.87 |  |

* SMC or placebo SMC was not co-administered with vaccines, and began approximately one month after dose 3 of the primary series, and the two boosters.

Table S3. Comparison of Geometric Mean Titers by sex of the child at different time points during the course of the study

| Contact | Sex | N | Geometric Mean (95% CI) | Ratio of Geometric Mean, Girls/Boys, (95% CI) | N with 2 fold increase in Titre | Prevalence Ratio (95% CI); Two-fold increase | P-value; Two-fold increase | N with 10 fold increase in Titre | Prevalence Ratio (95% CI); Ten-fold increase | P-value; Ten-fold increase |
| --- | --- | --- | --- | --- | --- | --- | --- | --- | --- | --- |
| Pre-2017 | Boys | 97 | 0.95 | NA |  |  |  |  |  |  |
|  | Girls | 104 | 0.95 | NA |  |  |  |  |  |  |
|  |  |  |  |  |  |  |  |  |  |  |
| Post-2017 | Boys | 97 | 396.5 (339.7, 462.8) | [Ref] | 96/97 | [Ref] |  | 96/97 | [Ref] |  |
|  | Girls | 101 | 344.2 (266.8, 444.1) | 0.87 (0.65, 1.17) | 98/104 | 0.97 (0.94, 1.00) | 0.084 | 98/104 | 0.97 (0.94, 1.00) | 0.084 |
|  |  |  |  |  |  |  |  |  |  |  |
| Pre-2018 | Boys | 147 | 44.4 (36.7, 53.7) | [Ref] |  |  |  |  |  |  |
|  | Girls | 132 | 40.3 (33.3, 48.8) | 0.91 (0.69, 1.19) |  |  |  |  |  |  |
|  |  |  |  |  |  |  |  |  |  |  |
| Post-2018 | Boys | 147 | 265.3 (234.1, 300.6) | [Ref] | 129/147 | [Ref] |  | 42/147 | [Ref] |  |
|  | Girls | 132 | 249.2 (216.2, 287.2) | 0.94 (0.78, 1.14) | 118/132 | 1.01 (0.93, 1.10) | 0.75 | 34/132 | 0.89 (0.61, 1.31) | 0.55 |
|  |  |  |  |  |  |  |  |  |  |  |
| Pre-2019 | Boys | 137 | 50.5 (42.1, 60.4) | [Ref] |  |  |  |  |  |  |
|  | Girls | 154 | 39.6 (33.5, 46.8) | 0.78 (0.61, 1.00) |  |  |  |  |  |  |
|  |  |  |  |  |  |  |  |  |  |  |
| Post-2019 | Boys | 137 | 202.0 (177.3, 230.1) | [Ref] | 114/137 | [Ref] |  | 14/137 | [Ref] |  |
|  | Girls | 154 | 158.1 (138.1, 180.9) | 0.78 (0.65, 0.94) | 132/154 | 1.03 (0.93, 1.13) | 0.60 | 17/154 | 1.05 (0.54, 2.03) | 0.89 |

Results combine children in both study countries, and in both intervention groups.

**Table S4**. Incidence of clinical malaria children among children in the two interventions groups who RTS,S/AS01_E_ combined according to post-vaccination anti-CSP antibody cut-off titer defined from reverse cumulative plots.

|  | PYAR | Events | Rate per 1000 PYAR (95% CI) | Hazard Ratio (95% CI) | Protective Efficacy (95% CI) |
| --- | --- | --- | --- | --- | --- |
| **2017** |  |  |  |  |  |
| Below threshold (266.8 EU/ml) | 52.7 | 11 | 208.6 (115.5, 376.7) | [Ref] | [Ref] |
| Above threshold | 131.9 | 6 | 45.5 (20.4, 101.2) | 0.21 (0.07, 0.58) | 79.5 (42.4, 92.7) |
|  |  |  |  |  |  |
| **2018** |  |  |  |  |  |
| Below threshold (207.2 EU/ml) | 102.0 | 29 | 284.3 (197.6, 409.1) | [Ref] | [Ref] |
| Above threshold | 175.6 | 42 | 239.2 (176.8, 323.7) | 0.89 (0.54, 1.45) | 11.3 (-44.8, 45.7) |
|  |  |  |  |  |  |
| **2019** |  |  |  |  |  |
| Below threshold (157.8 EU/ml) | 116.7 | 39 | 334.2 (244.2, 457.4) | [Ref] | [Ref] |
| Above threshold | 167.6 | 27 | 161.1 (110.5, 234.9) | 0.50 (0.29, 0.85) | 50.1 (15.0, 70.7) |
|  |  |  |  |  |  |
| **Overall*** |  |  |  |  |  |
| Below threshold | 271.4 | 79 | 291.1 (233.5, 362.9) | [Ref] | [Ref] |
| Above threshold | 475.1 | 75 | 157.9 (125.9, 198.0) | 0.60 (0.42, 0.84) | 40.4 (16.0, 57.7) |

The incidence of clinical malaria was compared between children with titers above and below a putative threshold protective titer, estimated from the reverse cumulative plots to be 266.8 EU/ml in 2017, 207.2 EU/ml in 2018 and !57.8 EU/ml in 2019. The threshold titer was defined as the minimum titer measured in X% of children who received RTS,S/AS01_E_, where X% was the efficacy of RTS,S against clinical malaria in that year of the study (ref 11). This was 71.7%, 63.2% and 58.6% in each year of the study, respectively.

*The overall analysis aggregates person time and events for the children above and below the specific threshold in each year of the study. The Cox regression models for the pooled analysis over all three years of the study were adjusted for study year and the age of the child.
